# Electromagnetic Control by Actuating Kirigami-Inspired Shape Memory Alloy: Thermally Reconfigurable Antenna application

**DOI:** 10.3390/s21093026

**Published:** 2021-04-26

**Authors:** Minjae Lee, Sukwon Lee, Sungjoon Lim

**Affiliations:** School of Electrical and Electronics Engineering, College of Engineering, Chung-Ang University, 221, Heukseok-Dong, Dongjak-Gu, Seoul 156-756, Korea; iamlmj720@gmail.com (M.L.); luxury231@hanmail.net (S.L.)

**Keywords:** electromagnetic control, kirigami-inspired, mechanical transformation, thermal actuator, shape memory alloy, reconfigurable antenna

## Abstract

Electromagnetic responses are generally controlled electrically or optically. However, although electrical and optical control allows fast response, they suffer from switching or tuning range limitations. This paper controls electromagnetic response by mechanical transformation. We introduce a novel kirigami-inspired structure for mechanical transformation with less strength, integrating a shape memory alloy actuator into the kirigami-inspired for mechanical transformation and hence electromagnetic control. The proposed approach was implemented for a reconfigurable antenna designed based on structural and electromagnetic analyses. The mechanical transformation was analyzed with thermal stimulus to predict the antenna geometry and electromagnetic analysis with different geometries predicted antenna performance. We numerically and experimentally verified that resonance response was thermally controlled using the kirigami-inspired antenna integrated with a shape memory alloy actuator.

## 1. Introduction

Electromagnetic control changes radio frequency (RF) component characteristics by controlling high-frequency alternating current. RF components and devices can be controlled to manipulate their mode change, allow reconfigurability, transform the shape transformation(s), etc. This reconfigurability can have multiple functions for a single component and reduces the number of components required for the RF system, increasing system gains and saving energy. Control is an active study topic in wireless communication, satellite communication, radar, and remote sensing, and RF components include sensors, filters, metasurfaces, and antennas [1,2,3,4,5,6].

Electromagnetic control can be achieved using electrical, optical, or magnetic methods [7,8]. An electrical component, such as pin [1,2,3,4,5,6] or varactor [6,9,10] diodes, have been widely employed to switch and control RF component operation. However, the required number of electronic components increases proportionally with the number of unit cells in a periodic structure, which can considerably impact the final cost, and also increases electromagnetic interference; a serious issue arises when designing electronic component DC biasing. Optical methods [11,12] have also been used similarly. Optical control is normally as fast as electrical, but the short wavelength makes it difficult to implement. Magnetic methods use magnetic material, e.g., ferrite, to control RF properties by controlling DC bias or magnetic field using a magnetic tensor [13,14]. However, magnetic approaches require bulky and difficult to control components. In general, the operating frequency is limited by parasitic capacitance. In addition, the active electrical devices suffer from nonlinearity at high power [15,16,17].

Electromagnetic control can also be achieved mechanically, modifying RF characteristics by mechanical transformation targeting parameters that affect RF properties [7,8]. Mechanical systems (using actuators, motors, etc. [18,19,20,21,22,23,24].) offer advantages from no operating frequency limitations to increased reliability in dust or moisture impacted environments compared with electrical and optical methods. In addition, the mechanical tuning range can be wider at high frequencies such as millimeter-wave or sub-terahertz spectrum [25]. However, mechanical approaches are generally more complex and costly to implement. Mechanical approaches using origami and kirigami structures have received increasing attention for solving this problem [26,27,28,29]. Origami, which means paper art in Japanese, has been employed in various fields using folding and unfolding structures. Kirigami adds cuts to basic origami structures and hence can implement more complex forms [26,27,28,29].

Several previous studies have proposed reconfigurable antennas using origami and kirigami structures [30,31,32,33,34]. For example, Shah et al. [31] proposed an origami quasi-Yagi helical antenna comprising three origami quasi-Yagi helical antennas that fold and unfold to act as driven, director, and reflector elements; and Lee et al. [32] proposed a frequency reconfigurable monopole antenna using kirigami techniques, varying the operating frequency by folded and unfolded three-story tower kirigami structural deformations. Shah et al. [33] proposed a deployable antenna utilizing kirigami pop-up geometry comprised of driven reflectors and a parasitic strip. Origami and kirigami structures allow mechanical transformation with small forces and can be implemented at a low cost.

This paper proposes mechanical, electromagnetic control using a shape memory alloy (SMA) actuator spring. SMA transforms into its original shape under external environmental stimulus, such as temperature [34]. Thus, the limited operating frequency can be overcome by using the spring as a thermal actuator, which also avoids adding noise, a common problem for motor-based mechanical control. We selected and analyzed a thermally reconfigurable antenna to demonstrate the proposed control. The proposed reconfigurable antenna was a kirigami-inspired structure that varies the operating frequency depending on its model. The proposed SMA actuator spring was transformed by applying a voltage to increase the temperature, changing between the circular patch and circular sector modes with correspondingly different resonance frequencies. In addition, this antenna can be used as a remote high-temperature alarm by modulating the frequency with high and low temperatures. The proposed control was proven numerically and experimentally using the SMA.

## 2. Electromagnetic Analysis for Proposed Antenna Design

**Figure 1:**
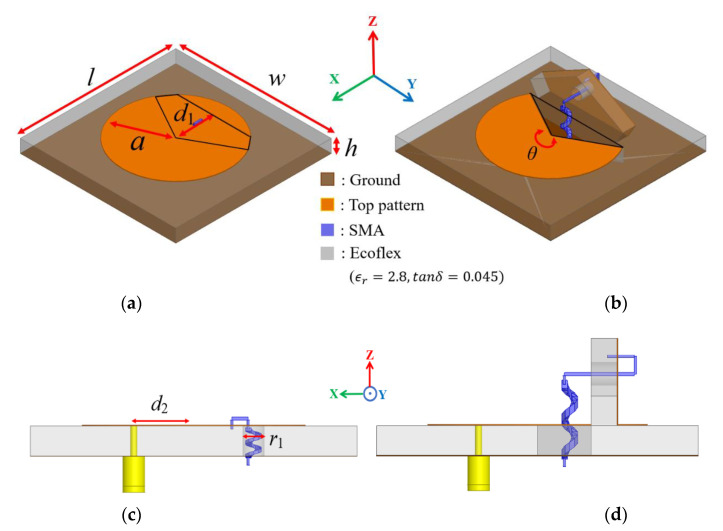
Proposed frequency reconfigurable kirigami-inspired antenna: perspective view in (**a**) circular disk and (**b**) circular sector modes; side view in (**c**) circular disk and (**d**) circular sector modes.

Figure 1 shows the proposed frequency reconfigurable kirigami-inspired antenna, designed using the ANSYS high-frequency structure simulator (HFSS). The antenna operates in circular disk mode when the SMA spring is pulled circular disk sector mode when the SMA spring is released. The circular antenna radius was determined from the cavity model [35,36]:

(1) $$a=\frac{K_{vm}c}{2\pi f_{r1}\sqrt{\epsilon_{r}}}=\frac{K_{11}c}{2\pi f_{r1}\sqrt{\epsilon_{r}}}$$

where $f_{r1}\;$is the resonant frequency of circular patch antenna, *c* is the speed of light, $\epsilon_{r}\;$ is the dielectric constant, $K_{vm}$ is the *m*th zero of derivative of the Bessel function of order *v*, and $K_{11}$ is the lowest value in circular patch antenna.

Here is the first Bessel’s function of order *v*:

(2) $$J_{v}\left(K_{vm}a\right)=0\{\begin{array}{c} \;\;\;v=n,\;\;\;\;\;if\;\theta =2\pi \\ \;v=\frac{n\pi }{\theta },\;\;\;\;\;\;otherwise \end{array}$$

where *a* is the circular patch antenna radius, *θ* is the ratio of the angle (expressed in radians), and *n* = 0,1,2,3….

From (1), it is possible to calculate the effective radius $a_{e}$:

(3) $$a_{e}={\left[a^{2}+\left(\frac{2ha}{\pi \epsilon_{r}}\right)\left(ln\frac{a}{2h}+\left(1.41\epsilon_{r}+1.77\right)+\frac{h}{a}\left(0.268\epsilon_{r}+1.65\right)\right)\right]}^{1/2}$$

where *h* is the substrate height.

Finally, the expected resonant frequency of the circular sector antenna equation is calculated with the following:

(4) $$f_{r2}=\frac{K_{vm}c}{2\pi a_{e}\sqrt{\epsilon_{e}}}=\frac{K_{61}c}{2\pi a_{e}\sqrt{\epsilon_{e}}}$$

where $f_{r2}$ is the resonant frequency of circular patch antenna, $\epsilon_{e}$ is the effective dielectric constant and $K_{61}$ is the lowest value when θ = 300° circular sector patch antenna.

We set the proposed antenna radius *a* = 20.5 mm, and length from the center (*d*_1_) was cut to implement the proposed kirigami-inspired structure. The circular patch was realized on an Ecoflex substrate with size *w* × *l* = 60 mm × 60 mm, thickness *h* = 5 mm, dielectric constant = 2.8, and tangential loss = 0.045. Both *w* and *l* correspond to 0.8λ_g_ at 2.4 GHz. Figure 1c,d show side views for each antenna mode with the SMA spring for mode transformation. The proposed antenna was fed by a coaxial probe and excited at distance *d*_2_ = 11 mm from the proposed antenna center for 50 Ω impedance matching. We allowed space *r*_1_ to insert the SMA spring, and the length was within the substrate height when the SMA was pulled.

**Figure 2:**
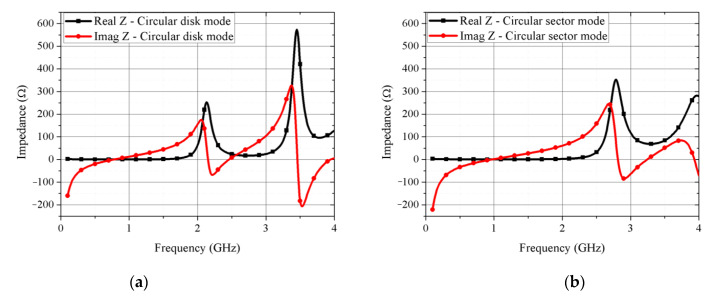
Simulated real and imaginary input impedance for the proposed antenna in (**a**) circular disk and (**b**) circular sector modes.

**Figure 3:**
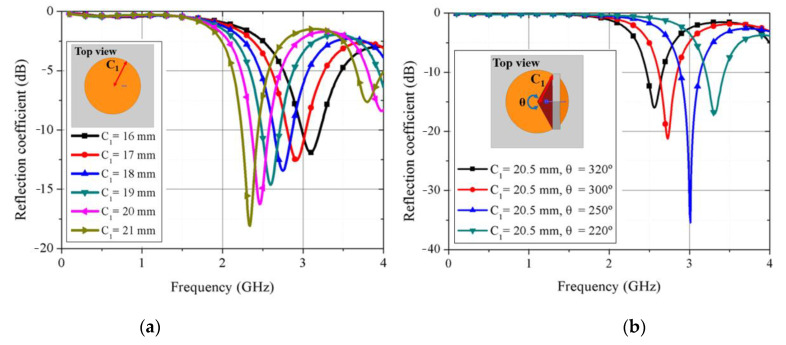
(**a**) Simulated reflection coefficient for circular disk mode (C_1_ = 16–21 mm); (**b**) simulated reflection coefficient for circular sector mode (C_1_ = 20.5 mm, θ = 220°, 250°, 280°, and 320°).

**Figure 4:**
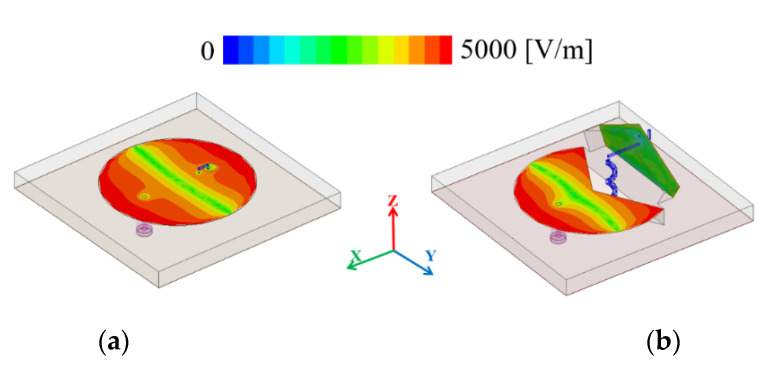
Electric field magnitude for (**a**) 2.41 GHz (circular disk mode) and (**b**) 3.25 GHz (circular sector mode).

**Figure 5:**
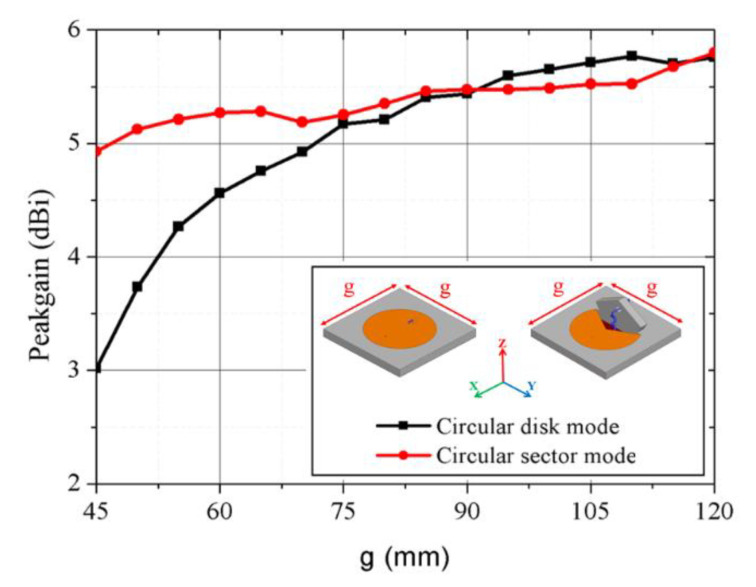
Comparison of antenna peak gain according to ground size.

Figure 2 shows that simulated real and imaginary input impedances for the circular disk and circular sector modes change according to the mode conversion. Figure 3 shows the simulation results of the proposed kirigami-inspired antenna. As shown in Figure 3a, when the radius (C_1_ = 16 to 21 mm) of circular disk mode is larger, the resonant frequency is decreased. As shown in Figure 2b, when the fixed radius (C_1_ = 20.5 mm) with a longer arc length (larger θ = 220°, 250°, 280°, and 320°) of the major sector, the resonant frequency is decreased, as well. Figure 4a,b show electric field magnitude for the circular disk and circular sector modes at mode at 2.41 and 3.25 GHz, respectively. The circular disk mode exhibited a large electric field at the antenna edges (Figure 4a), whereas the circular sector mode exhibited a reduced electric field in regions where the electric field gathers due to the structural change. Figure 5 shows the comparison of the proposed antenna peak gain according to ground size. The peak gain was increased with a larger ground size (Figure 5). In this paper, we set the ground size at 60 mm for moderate peak gain and easy fabrication.

## 3. Structural Analysis for Thermal Reconfigurability

**Figure 6:**
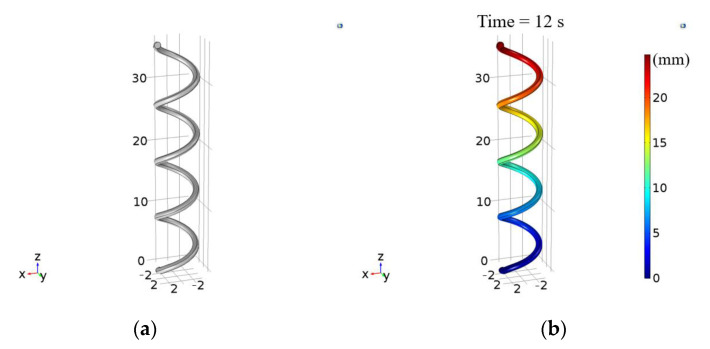
(**a**) SMA spring rendered in COMSOL and corresponding (**b**) simulated total displacement.

Structural analysis simulations were performed using COMSOL Multiphysics for the kirigami-inspired structure and SMA spring. Figure 6a shows the final design incorporating the SMA spring. Material properties were selected for Ti and Ni using standard COMSOL Multiphysics modules, with −2.7 × 10^−4^ K^−1^ coefficient of thermal expansion obtained by optimizing the SMA spring experiment results from [37] using COMSOL. The ambient temperature for this simulation was set at 253.15 K. Figure 6b shows SMA spring total displacement after 12 s heating from that paper. Similar results were obtained for the SMA spring used in the current paper.

**Figure 7:**
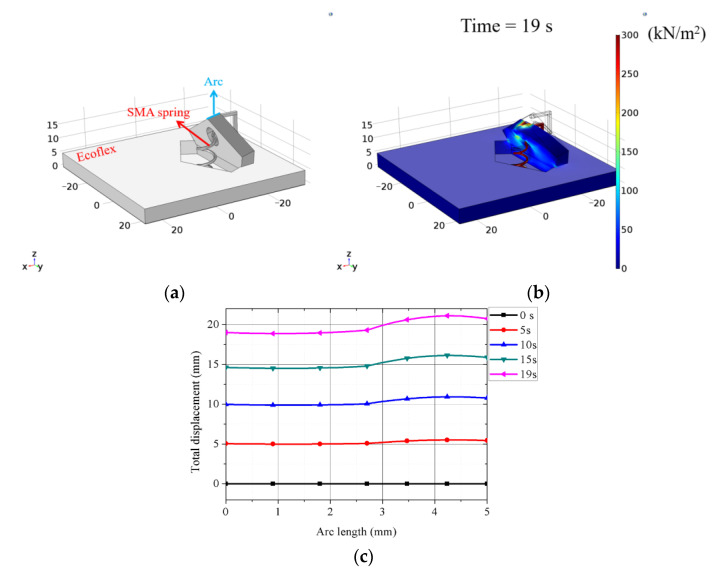
(**a**) Kirigami-inspired structure with SMA rendered in COMSOL; (**b**) simulated von Mises stress; (**c**) total displacement with respect to time.

Figure 7a shows the kirigami-inspired structure design with SMA spring in COMSOL. SMA spring properties were the same as simulated in Figure 6, and Ecoflex 00–30 parameters were Young’s modulus = 125 kPa and Poisson’s ratio = 0.49 [38,39]. Figure 7b shows simulated von Mises stress from applying 0.68 V at 3 A to the SMA spring for 19 s. Thus, 270 kN/m^2^ was applied to the connection between the kirigami-inspired structure and the SMA spring. Figure 7c shows total displacement with respect to time for the arc shown in Figure 7a. Simulated results for arc displacement suggest 16 s to convert circular sector to circular disk mode.

## 4. Fabrication and Experimental Demonstration

**Figure 8:**
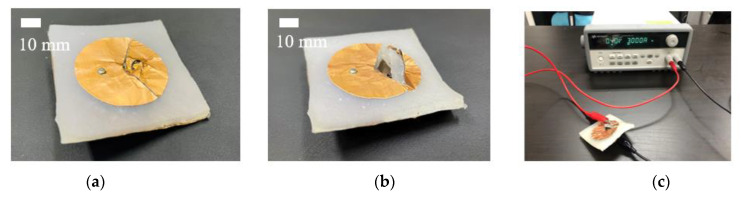
Fabrication sample in (**a**) circular disk and (**b**) circular sector modes. (**c**) Measurement setup.

**Figure 9:**
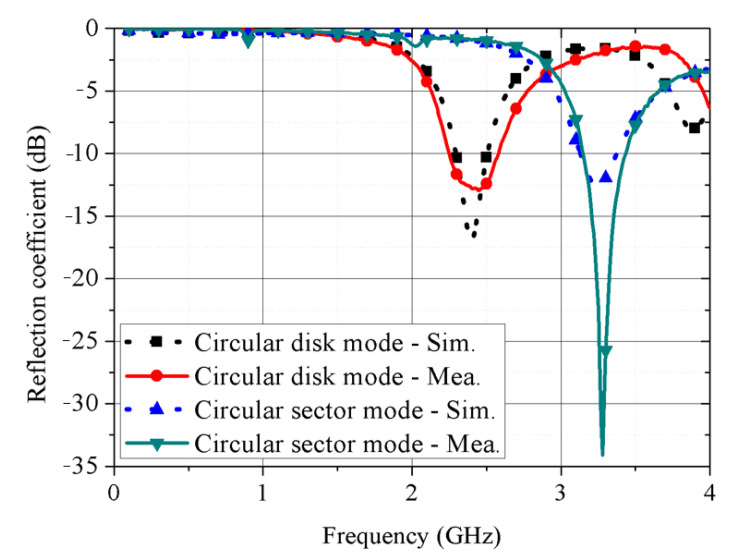
Simulated and measured reflection coefficient for a proposed antenna in circular disk mode and circular sector mode.

Figure 8 shows the proposed antenna fabricated sample to experimentally verify the concept. Figure 8a,b shows the circular disk and circular sector modes. The circular patch conductive pattern was implemented using copper tape. Ecoflex 00-30 was provided by Smooth-On, Inc (5600 Lower Macungie Road, Macungie, PA 18062, USA). The SMA spring employed was a commercial product, with 150 mm total length and 9 mm pitch. However, we only used a 5 mm section for the proposed kirigami-inspired structure. The spring was one-way with 10 N force (nominal) when pulled. Figure 8c shows that the SMA spring was pulled within 16–20 s by applying 0.68 V at 3 A was applied, confirming successful mode conversion. Figure 9 compares simulated and measured reflection coefficient for the proposed antenna in the circular disk and circular sector modes. The reflection coefficient was measured with an Anritsu MS2038C network analyzer. Simulated and measured reflection coefficient = −17.12 and −12.81 dB at 2.4 GHz, and −12.65 and −22.43 dB at 3.25 GHz for the circular disk and circular sector modes, respectively.

**Figure 10:**
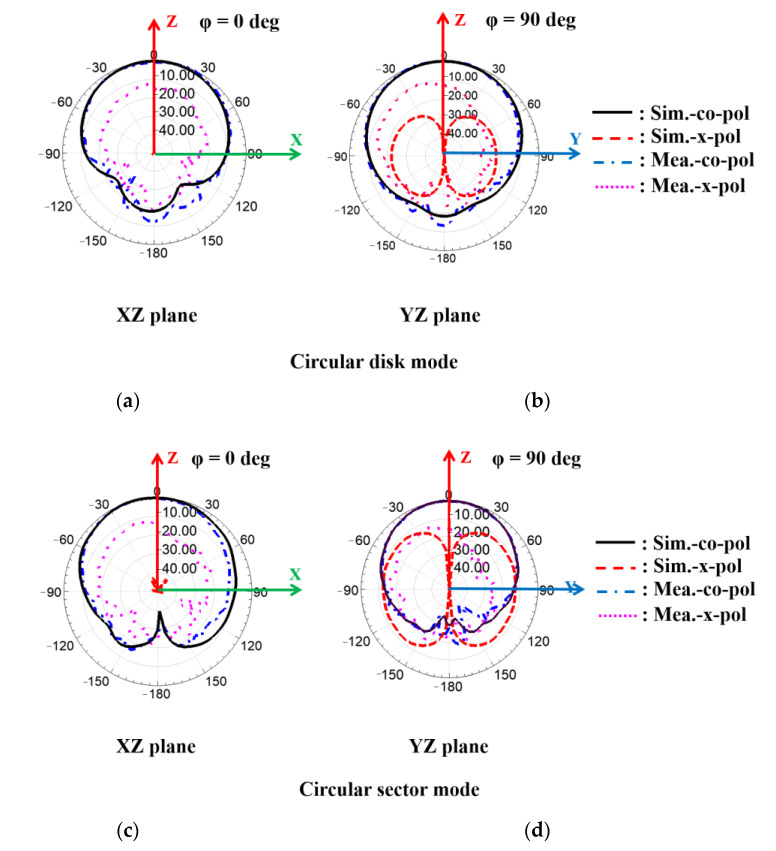
Simulated and measured normalized radiation patterns for circular disk mode in the (**a**) *XZ* and (**b**) *YZ* planes; and circular sector mode in the (**c**) *XZ* and (**d**) *YZ* plane.

**Figure 11:**
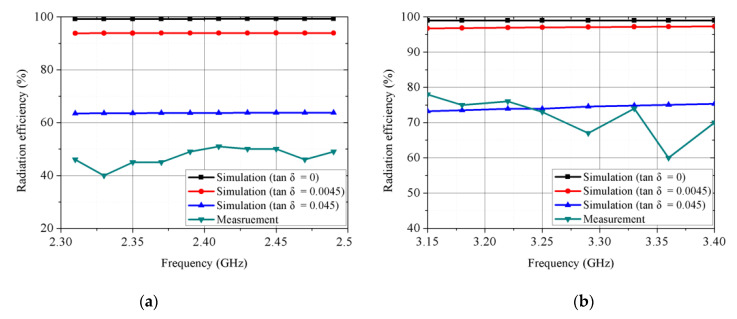
Simulated and measured radiation efficiency at different tangential loss: (**a**) circular disk mode and (**b**) circular sector mode.

Figure 10 shows simulated and measured normalized radiation patterns for the proposed antenna for each mode. Figure 10a,b show**s** normalized radiation patterns in *XZ* and *YZ* planes, respectively, in circular disk mode at 2.41 GHz, with simulated and measured peak gains = 4.9 and 4.26 dBi, respectively. Figure 10c,d show**s** normalized radiation patterns in *XZ* and *YZ* planes, respectively, in circular sector mode at 3.25 GHz, with simulated and measured peak gains = 5.95 and 6.41 dBi, respectively. The measured difference between co and cross-polarization = 15.49 and 18.47 dB in the boresight direction in the circular disk and circular sector modes, respectively. Figure 11a,b show**s** the simulated and measured radiation efficiency at different tangential losses for the circular disk mode and circular sector mode, respectively. Simulated and measured radiation efficiency = 65.55 and 51.44% at 2.41 GHz and 76.42 and 75.5% at 3.25 GHz for circular disk and circular sector modes, respectively. As shown in Figure 11, the radiation efficiency can be increased by decreasing the tangential loss of the substrate. In addition, the simulated efficiency is compared with the measured efficiency. The difference between the simulated and measured efficiency was due to the adhesive film from the copper tape.

In Table 1, the proposed antenna was compared with other frequency reconfigurable antennas with different tuning technologies. It was observed that the mechanical and thermal tuning methods could achieve a wider frequency tuning range compared to electrical and optical tuning methods because of their low parasitic capacitance. In this work, we sacrificed the tuning range for less stress of the SMA spring. Nevertheless, its tuning range can be increased with a longer SMA spring or larger mechanical deformation.

## 5. Discussion

The proposed concept could be used to advance smart structure and actuator technology. Although the SMA springs used here are one-way, i.e., they can only be changed in one direction, two-way springs would enable two-way variation. In addition, we used copper tape for a fast demonstration of the proposed antenna. It was possible because of simple conductive patterns. For complicated conductive patterns and robustness, the conductive patterns can be realized by additive manufacturing technology such as inkjet printing [45,46], aerosol jet printing [47], or screen printing [48].

This antenna can be used as the multiple-input multiple-output (MIMO) antenna. We investigated the MIMO spatial diversity by simulating two array antennas. MIMO systems can obtain high capacity by using multiple antennas. However, minimizing interference between antennas is one of the important factors for MIMO, as coupling between multiple antennas can lead to poor performance. [49] For the MIMO system, the envelope correction coefficient (ECC) is an important parameter indicating the interference between antennas.

The ECC can be calculated as follows [50]:

(5) $$\rho_{e}=\frac{{\left|S_{11}{}^{*}S_{12}+S_{12}{}^{*}S_{22}\right|}^{2}}{\left(1-{\left|S_{11}\right|}^{2}-{\left|S_{21}\right|}^{2}\right)\left(1-{\left|S_{22}\right|}^{2}-{\left|S_{12}\right|}^{2}\right)}$$

**Figure 12:**
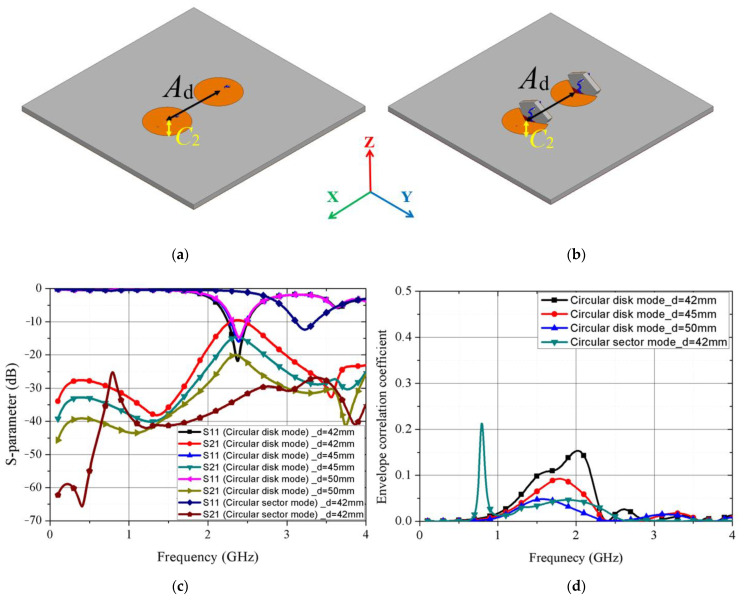
MIMO arrangement of the proposed antenna (**a**) at circular disk mode, and (**b**) at circular sector mode; (**c**) simulated s-parameter and (**d**) envelope correlation coefficient.

Figure 12a,b shows the distance (*A*_d_) between the centers of the two array antennas for each mode. The Radius (C_2_) of the array antenna in each mode is 20.5 mm, and the S-parameter when the distance (*A*_d_) between the antennas was changed to 42, 45, and 50 mm is shown in Figure 12c. In general, the MIMO system has good isolation if the mutual coupling level (*S*_21_) is lower than −15 dB. [49] When the distance *(A*_d_) between two antenna elements was larger than 45 mm, S_21_ was lower than −15 dB. Figure 12d shows the calculated ECC from Equation (5). The ECC value when the distance (*A*_d_) is 42 mm was 0.0004 for the circular disk mode at 2.41 GHz, and the circular sector mode is 0.019 at 3.25 GHz, respectively. The MIMO arrangement of the proposed antenna had good diversity performance for a MIMO antenna. [49]

## 6. Conclusions

This paper proposes a kirigami-inspired antenna that can be transformed mechanically to change its electromagnetic response using an SMA actuator spring. The proposed kirigami-inspired antenna was comprised of a flexible Ecoflex substrate and SMA spring that could mechanically transform from circular disk to circular sector mode by pulling and releasing the SMA spring. The proposed antenna could vary resonance frequency from 2.41 to 3.25 GHz. We numerically and experimentally demonstrated that the resonance response was thermally controlled by actuating the SMA.

## Figures and Tables

**Table 1:** Comparison table of frequency reconfigurable antennas with different tuning technologies.

Ref.	Tuning Tech.	Method	Tuning Range (%)	Size (mm) (W × L × H)	Gain (dBi)	Efficiency (%)	DC Biasing Circuit	Complexity	Cost
[40]	Electrical	Pin-diode	49	127 × 127 × 2.54	−1.1	47	Yes	High	High
[41]	Electrical	Varactor-diode	25	150 × 150 × 1.524	N/A	N/A	Yes	High	High
[11]	Optical	Photoconductive switch	25	4.56 × 48.94 × N/A	8–9	N/A	No	High	High
[42]	Mechanical	Microfluidic	70	100 × 100 × 3.175	6.98–7.34	80.5–88.0	No	Low	Low
[43]	Mechanical	MEMS switch	149	45 × 41.8 × 7.126	1.2–3.3	75–85	No	High	Low
[44]	Thermal	SMA	148	60 × 50 × 1.6	−17.13–3.18	N/A	No	Low	Low
This work	Thermal	SMA spring	30	60 × 60 × 5	4.26–6.41	51.44–75.5	No	Low	Low

## Data Availability

The data presented in this study are available within the article.
